# Supplementary material for: A combinatorial action of GmMYB176 and GmbZIP5 controls isoflavonoid biosynthesis in soybean (Glycine max)
Source: Commun Biol. 2021 Mar 19;4:356. doi: 10.1038/s42003-021-01889-6 (PMC7979867; doi:10.1038/s42003-021-01889-6)
Supplement: Supplementary file 2 — Supplementary Information [file 42003_2021_1889_MOESM2_ESM.pdf]

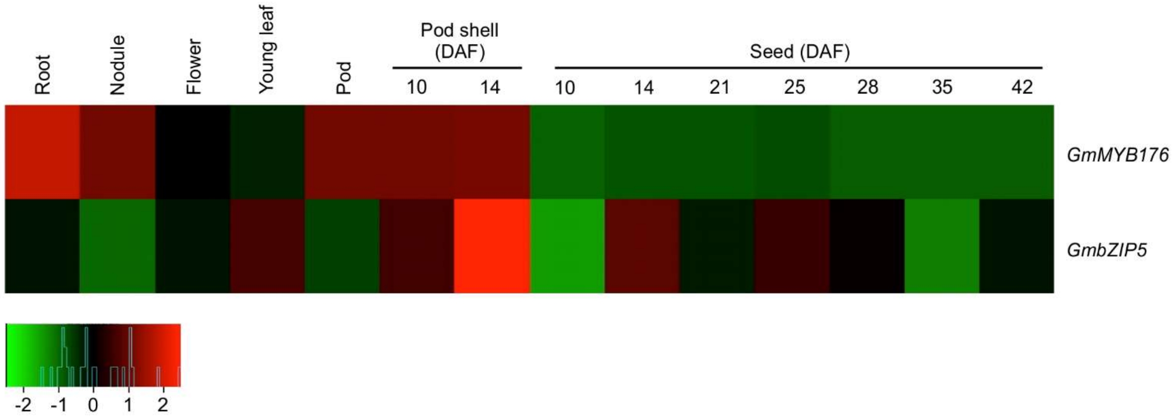

Supplementary Fig. 1. Expression of GmMYB176 and GmbZIP5 in developing soybean seeds. The transcriptome data were retrieved from SoyBase database (https://soybase.org/) for heatmap generation. Low and high transcript abundances are indicated by green and red color, respectively in the color scale.

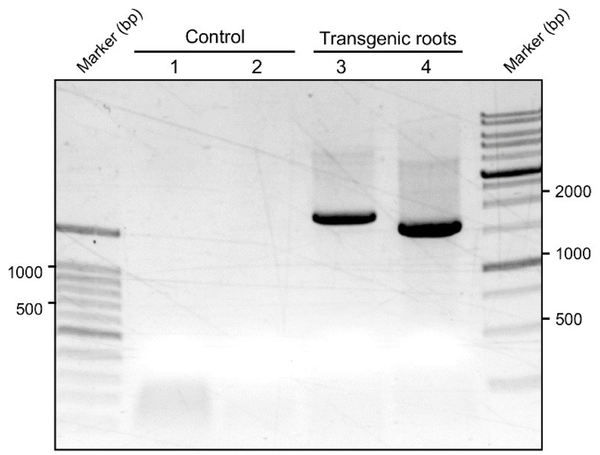

Supplementary Figure 2. Expression of *GmMYB176-GmbZIP4* and *GmMYB176-GmbZIP5* in transgenic and control soybean hairy root tissues. Total RNA (1 µg) extracted from soybean hairy roots overexpressing *GmMYB176-GmbZIP4* or *GmMYB176-GmbZIP5* or control was used for reverse transcriptase-polymerase chain reaction (RT-PCR) with GmMYB176-specific forward and GmbZIP4 or GmbZIP5-specific reverse primers. The expected size of the fusion genes are 1764 bp (*GmMYB176-GmbZIP4*) and 1548 bp (*GmMYB176-GmbZIP5*). cDNA synthesized from control hairy roots used as a template in RT-PCR using GmMYB176-specific forward and GmbZIP4-specific reverse primers (Lane 1), GmMYB176-specific forward and GmbZIP5-specific reverse primers (Lane 2), cDNA synthesized from *GmMYB176-GmbZIP4* overexpressing hairy roots used as a template in RT-PCR using GmMYB176-specific forward and GmbZIP4-specific reverse primers (Lane 3) and cDNA synthesized from *GmMYB176-GmbZIP5* overexpressing hairy roots used as a template in RT-PCR using GmMYB176-specific forward and GmbZIP5-specific reverse primers (Lane 4) are shown.

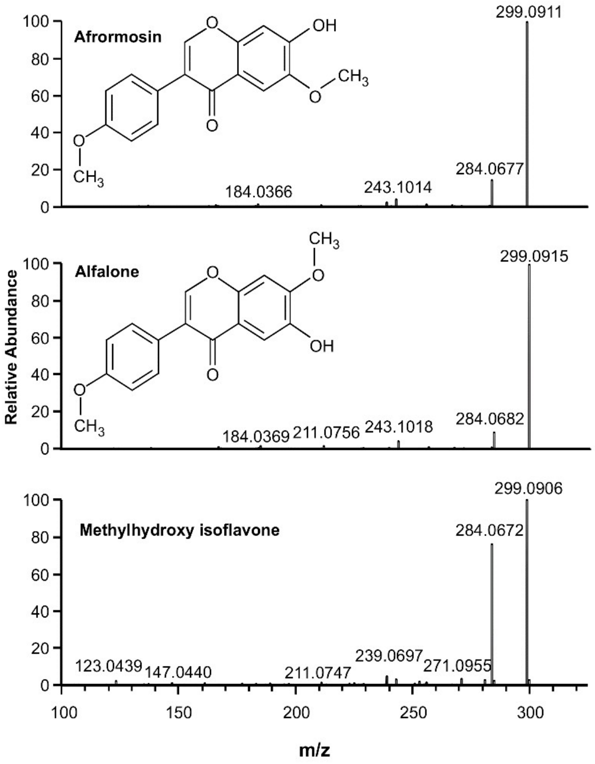

Supplementary Figure 3. MS/MS fragmentation [M+H] 35kV normalized collision energy of afrormosin, alfalone, and O-methylhydroxy isoflavone. Comparison of MS/MS fragmentation of O-methylhydroxy isoflavone identified in GmMYB176-GmbZIP5 overexpressing roots against the standards indicate the detected compound is not afrormosin or alfalone.
