# Supplementary material for: A combinatorial action of GmMYB176 and GmbZIP5 controls isoflavonoid biosynthesis in soybean (Glycine max)
Source: Commun Biol. 2021 Mar 19;4:356. doi: 10.1038/s42003-021-01889-6 (PMC7979867; doi:10.1038/s42003-021-01889-6)
Supplement: Supplementary file 3 — Description of Additional Supplementary Files [file 42003_2021_1889_MOESM3_ESM.pdf]

Description of Additional Supplementary Files

File name: Supplementary Data 1

Description: The list of 716 candidates identified in Co-IP using GmMYB176-YFP and YFP-GmMYB176 as baits.

File name: Supplementary Data 2
Description: Regulatory element (RE) sites on the GmCHS8-30bp fragment of GmCHS8 gene promoter.

File name: Supplementary Data 3
Description: List of metabolite features identified in GmMYB176-GmbZIP5 overexpressing roots by LC-MS/MS (ESI+).

File name: Supplementary Data 4
Description: Sequence of oligonucleotides used for gene amplification.

File name: Supplementary Data 5
Description: Source data for main Figures.
