# Supplementary material for: A combinatorial action of GmMYB176 and GmbZIP5 controls isoflavonoid biosynthesis in soybean (Glycine max)
Source: Commun Biol. 2021 Mar 19;4:356. doi: 10.1038/s42003-021-01889-6 (PMC7979867; doi:10.1038/s42003-021-01889-6)
Supplement: Supplementary file 9 — Reporting Summary [file 42003_2021_1889_MOESM9_ESM.pdf]

nature research

Corresponding author(s): Sangeeta Dhaubhadel

Last updated by author(s): Feb 2, 2021

Reporting Summary

Nature Research wishes to improve the reproducibility of the work that we publish. This form provides structure for consistency and transparency in reporting. For further information on Nature Research policies, see our Editorial Policies and the Editorial Policy Checklist.

Statistics

For all statistical analyses, confirm that the following items are present in the figure legend, table legend, main text, or Methods section.

n/a	Confirmed	
☐	☒	The exact sample size (*n*) for each experimental group/condition, given as a discrete number and unit of measurement
☒	☐	A statement on whether measurements were taken from distinct samples or whether the same sample was measured repeatedly
☐	☒	The statistical test(s) used AND whether they are one- or two-sided<br>*Only common tests should be described solely by name; describe more complex techniques in the Methods section.*
☒	☐	A description of all covariates tested
☒	☐	A description of any assumptions or corrections, such as tests of normality and adjustment for multiple comparisons
☐	☒	A full description of the statistical parameters including central tendency (e.g. means) or other basic estimates (e.g. regression coefficient) AND variation (e.g. standard deviation) or associated estimates of uncertainty (e.g. confidence intervals)
☐	☒	For null hypothesis testing, the test statistic (e.g. *F*, *t*, *r*) with confidence intervals, effect sizes, degrees of freedom and *P* value noted<br>*Give P values as exact values whenever suitable.*
☒	☐	For Bayesian analysis, information on the choice of priors and Markov chain Monte Carlo settings
☒	☐	For hierarchical and complex designs, identification of the appropriate level for tests and full reporting of outcomes
☒	☐	Estimates of effect sizes (e.g. Cohen's *d*, Pearson's *r*), indicating how they were calculated

Our web collection on statistics for biologists contains articles on many of the points above.

Software and code

Policy information about availability of computer code

Data collection	N/A
Data analysis	For co-IP analysis, MS/MS data were analyzed by MassLynx 4.1 software with Mascot (http://www.matrixscience.com) and compared against the soybean protein database in Phytozome.<br>qPCRdata were analyzed using Bio-Rad CFX Maestro (Bio-Rad, USA).<br>For the metabolomics analysis and alignment of the detected peaks, the XCMS package in R was used as described by Gracia et al (2020) with the addition of diffreport-method to create summary report. Compounds that showed differential accumulation were chosen for further identification through Xcalibur software.

For manuscripts utilizing custom algorithms or software that are central to the research but not yet described in published literature, software must be made available to editors and reviewers. We strongly encourage code deposition in a community repository (e.g. GitHub). See the Nature Research guidelines for submitting code & software for further information.

Data

Policy information about availability of data

All manuscripts must include a data availability statement. This statement should provide the following information, where applicable:

- Accession codes, unique identifiers, or web links for publicly available datasets
- A list of figures that have associated raw data
- A description of any restrictions on data availability

All data generated or analyzed during this study are included in the article, supplementary data, or provided with identifiers/URLs or available from authors on reasonable request.

April 2020

Field-specific reporting

Please select the one below that is the best fit for your research. If you are not sure, read the appropriate sections before making your selection.

☒ Life sciences ☐ Behavioural & social sciences ☐ Ecological, evolutionary & environmental sciences

For a reference copy of the document with all sections, see nature.com/documents/nr-reporting-summary-flat.pdf

Life sciences study design

All studies must disclose on these points even when the disclosure is negative.

Sample size	For co-IP experiments, transgenic hairy roots were collected from multiple independent transgenic events and pooled in groups. A total of six soybean hairy root samples (2 YFP tagged bait proteins; 3 replicates each) were used.<br>For qPCR and metabolomic analysis, samples were collected as described above and a total of 10 biological replicates were used. Each replicate is a pool of soybean hairy roots and each hairy roots is an individual transgenic event.
Data exclusions	No data were excluded.
Replication	Three replicates of each YFP tagged proteins baits were used in the co)IP experiments. Each replicate was processed independently using soybean hairy roots. The aim was to identify all possible GmMYB176 interactors with two different YFP tagged baits and 3 replicates each.For qPCR and metabolomic analyses, samples were collected as described above and a total of 10 biological replicates were used.
Randomization	This is not relevant to our study. For example, in co-IP, 2 baits with 3replicates each were used. Each replicate was a pool of transgenic soybean hairy roots that can be considered as a population.
Blinding	This is not relevant to our study as the samples were soybean hairy roots. Each sample group contained known treatment/condition/ transgene. For example, GmMYB176 was either silenced or over-expressed compared to the control hairy roots for metabolomic study.

Reporting for specific materials, systems and methods

We require information from authors about some types of materials, experimental systems and methods used in many studies. Here, indicate whether each material, system or method listed is relevant to your study. If you are not sure if a list item applies to your research, read the appropriate section before selecting a response.

Materials & experimental systems

n/a	Involved in the study	
☐	☒	Antibodies
☒	☐	Eukaryotic cell lines
☒	☐	Palaeontology and archaeology
☒	☐	Animals and other organisms
☒	☐	Human research participants
☒	☐	Clinical data
☒	☐	Dual use research of concern

Methods

n/a	Involved in the study	
☒	☐	ChIP-seq
☒	☐	Flow cytometry
☒	☐	MRI-based neuroimaging

Antibodies

Antibodies used	Living Colors® A.v. (anti-GFP) Monoclonal Antibody (Clontech, USA) Cat#632381, lot# 1004037.
Validation	Quality Control Data from the supplier:<br>The quality and performance of this lot of Living Colors A.v. Monoclonal Antibody (JL-8) was tested by Western blot analysis using lysate made from a HEK 293 cell line stably expressing AcGFP1. After cells were collected and lysed using SDS sample buffer, the lysate (10 μl; equivalent to 35,000 cells) was electrophoresed on a 12% SDSpolyacrylamide gel and transferred to a nitrocellulose membrane. The blot was probed with the Living Colors A.v. Monoclonal Antibody, JL-8 (diluted 1:1,000), followed by a secondary goat anti-mouse antibody conjugated to horseradish peroxidase (HRP). The HRP signal was detected by chemiluminescence. A band of approximately 30 kDa corresponding to AcGFP1 was observed in the lane loaded with the AcGFP1 cell lysate. A band of this size was not detected in the lysate of untransfected HEK 293 cells.<br>Reference: DOI: 10.1038/ncomms11379<br>We have also used it previously in the following studies:<br>- Dastmalchi, M., Bernards, M. and Dhaubhadel, S.(2016) Twin anchors of the soybean isoflavonoid metabolon: IFS and C4H tether the complex to the endoplasmic reticulum. Plant Journal, 85, 689-706<br>- Sepiol, C.J., Yu, J. and Dhaubhadel, S.(2017) Genome-wide identification of chalcone reductase gene family in soybean: insight into root-specific GmCHRs and Phytophthora sojae resistance. Frontiers in Plant Science, doi: 10.3389/fpls.2017.02073
